# Non-functional retroperitoneal paraganglioma: A report of case with literature review

**DOI:** 10.1016/j.amsu.2021.102360

**Published:** 2021-04-28

**Authors:** Amal Hajri, Ahmed Ballati, Zakaria Essaidi, Driss Errguibi, Rachid Boufettal, Saad Rifki El Jai, Farid Chehab

**Affiliations:** a Department of General Surgery, University Hospital Centre Ibn Rochd, Casablanca, Morocco; b Faculty of Medecine and Pharmacy, Hassan II University, Casablanca, Morocco

**Keywords:** Paraganglioma, Diagnosis, Surgery

## Abstract

**Introduction:**

Retroperitoneal paragangliomas are rare tumors, they arise from ganglia along the sympathetic and parasympathetic chain. We report a rare case of a non functional paraganglioma in whom surgical resection was performed.

**Presentation of case:**

A 35 years-old female presented with chronic abdominal pain, A contrast magnetic resonance imaging (MRI) of abdomen showed a well-defined Left latero-aortic cystic retro-peritoneal surgical resection using laparotomy was performed, The patiente recovered well and was discharged three days after surgery. Histological examination and immunohistochemical revealed a retroperitoneal paraganglioma.

**Discussion and conclusion:**

Non-functioning retroperitoneal paragangliomas are rare and are most often Isolated. Radiological techniques including, Contrast-enhanced computed tomography (CT) and Magnetic resonance imaging (MRI) are useful for identifying and locating retroperitoneal paragangliomas. surgical excision is still the most effective treatment when it possible.

## Highlights

• We report a case of non-functional retroperitoneal paraganglioma.
• Non-functional retroperitoneal paraganglioma is extremely rare and mostly benign.
• A difficult tumour to diagnose and treat.
• Complete surgical excision is the treatment of choice for extra-adrenal paragangliomas.

## 1 Introduction

Retroperitoneal paragangliomas are rare tumors, While pheochromocytomas originate in the core of the adrenal glands, extra-adrenal paraganglioma tumors arise from ganglia along the sympathetic and parasympathetic chain. Most of them are functional, with symptoms and signs of catecholamine overproduction, similar to pheochromocytoma except for the variation in the anatomic location The risk of malignancy may be as high as 20%–30% compared with a 10% risk of malignancy for adrenal pheochromocytoma [1].

We report a rare case of a non functional paraganglioma in whom surgical resection was performed. This work has been reported in line with the SCARE criteria [2].

## 2 Case presentation

**Fig. 1:**
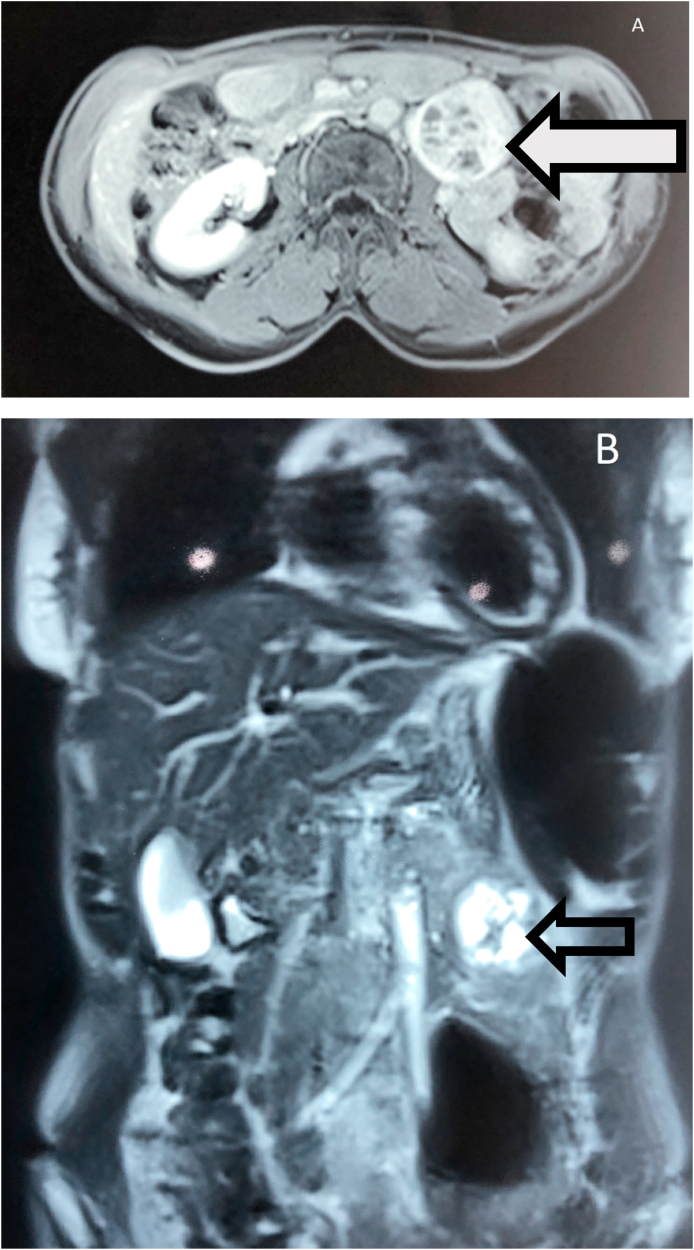
(A, B) Contrast magnetic resonance imaging (MRI) of the abdomen (axial and coronal planes) revealing a well-defined Left latero-aortic cystic retro-peritoneal mass (left arrow).

**Fig. 2:**
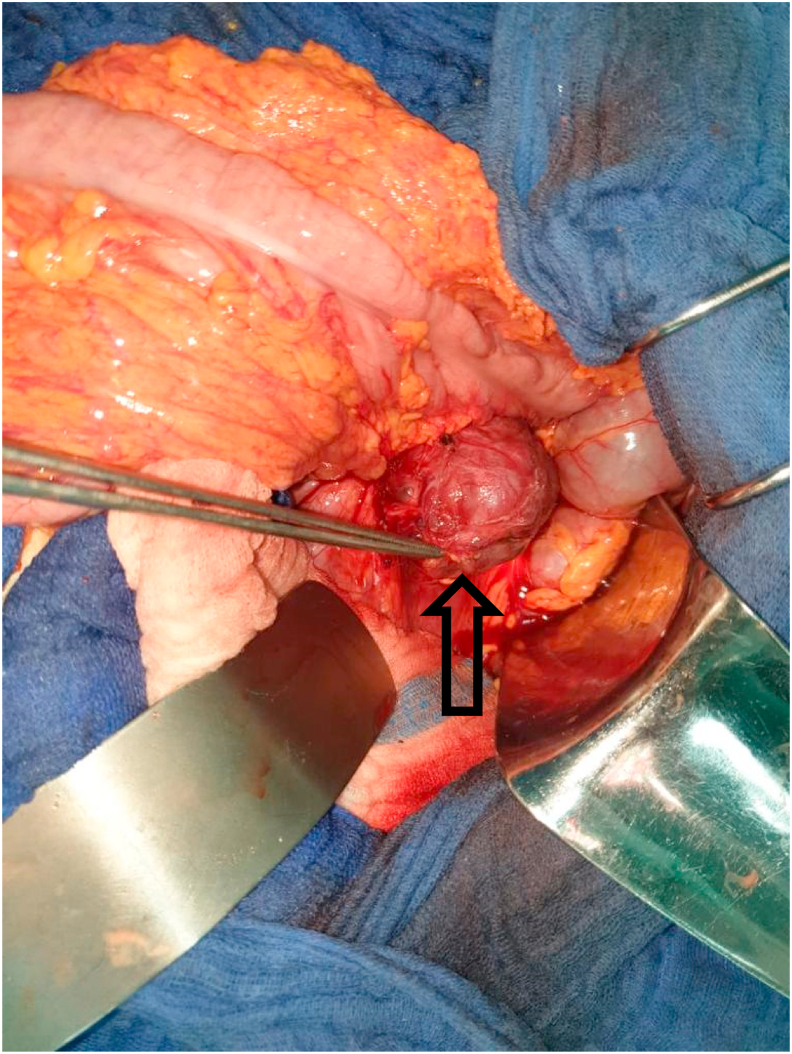
Intraoperative photograph showing the presence of a 6 cm Retroperitoneal cystic mass (up arrow).

**Fig. 3:**
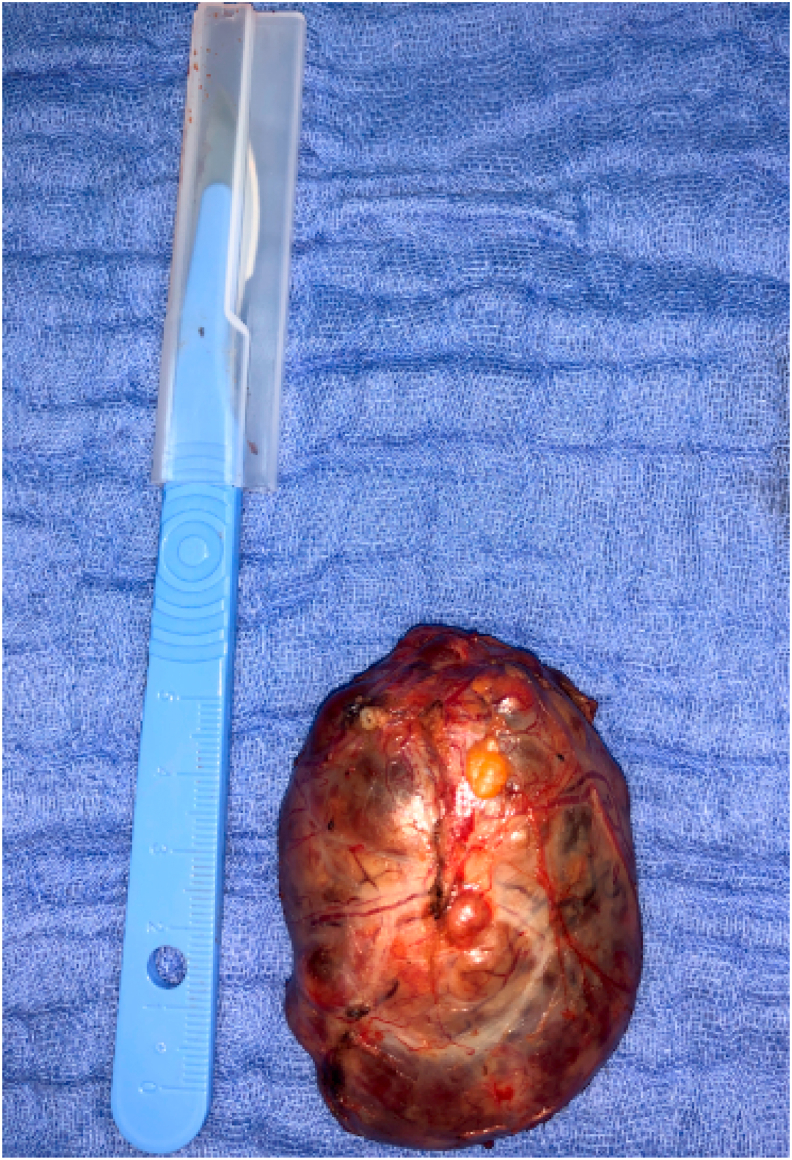
Specimen.

A 35 years-old female with a medical history of chronic abdominal pain for about four months was admitted to our hospital Abdominal examination was normal.Abdominal and pelvis Ultrasound (US) revealed a Left retro peritoneal formation measuring 4cm rounded well-defined with heterogeneous non-vascularized content. A contrast magnetic resonance imaging (MRI) of abdomen showed a well-defined Left latero-aortic cystic retro-peritoneal mass measuring 45 × 50 mm appear as hypointense in T1w and hyperintense in T2w sequence with multiple septa (Fig. 1). Biochemical detection included measurement of plasma catecholamine concentration and vanillylmandelic acid (VMA) in a 24-h urine collection sample were within normal values. she underwent surgical resection under general anesthesia using laparotomy, the exploration of the left anterior pararenal space confirmed the presence of a 6 cm cystic mass (Fig. 2) that was carefully resected (Fig. 3). The patiente recovered well and was discharged Three days after surgery. Histological examination and immunohistochemical revealed a retroperitoneal paraganglioma.

## 3 Discussion

Paraganglioma (PG), also known as extra-adrenal pheochromocytoma, is a chromaffin cell tumor located at various sites along the sympathetic and parasympathetic chain, they can thus be found in the head, neck, thorax, abdomen, and pelvis. Retroperitoneal paragangliomas are rare tumors. Non-functioning retroperitoneal forms are even more rare and are most often isolated [3].

Histologically, most are composed of well-differentiated neuroendocrine cells disposed in small clusters (zellballen) or cords separated by prominent fibrovascular stroma. Distinctive within the cells in most tumors are dark neurosecretory granules that contain catecholamines. Sometimes the cells are spindle shaped. Mitoses are usually infrequent, but occasional tumors are overtly anaplastic and pleomorphic and contain numerous mitoses. The malignant potential is determined by local invasion and also distant metastases [4].

Functional PGs can be diagnosed based on clinical presentation, the most common type of presenting symptom were associated with catecholamine-secreting paragangliomas: Episodic headache, diaphoresis and tachycardia. Episodic hypertension has been identified as a characteristic feature of catecholamine-secreting paragangliomas and is used for the differential diagnosis of paragangliomas, laboratory examination showing elevated catecholamines and their metabolites (VMA) in the blood and urine. Nonfunctional PGLs are mostly found incidentally or through symptoms caused by compression of the surrounding organs [5]. In our case the symptomatology was made of chronic abdominal pain.

Radiological techniques including Ultrasound, Contrast-enhanced computed tomography (CT) and Magnetic resonance imaging (MRI) are useful for identifying and locating retroperitoneal paragangliomas, the imaging characteristics of retroperitoneal paragangliomas included soft-tissue masses in the sympathetic chains associated with the abdominal aorta, cystic degeneration and necrosis inside the masses functional imaging, such as PET scanning and MIBG scintigraphy may help detect primary or metastatic tumors that could be missed on CT/MRI [6], in our case (MRI) scan showed a well-defined Left latero-aortic cystic retro-peritoneal mass measuring 45 × 50 mm with multiple septa.

A complete surgical resection is still the most effective and the only potentially curative treatment and should be attempt even in patients with distant metastasis.

Open exploration and resection is the standard surgical management of PG, however, laparoscopic resection of PG is considered challenging because of the altered anatomic location, dense peritumoral adhesions, high vascularity envelope, proximity to major blood vessels [1,7]. If a tumor is felt to be unresectable at surgery, attempts to reduce its size by chemotherapy or radiation or embolization may be indicated because resection offers the only chance of cure [4]. In this case a complete surgical resection using laparotomy were performed. Histological examination of the mass revealed a retroperitoneal paraganglioma.

Patient with metastatic disease will require adjuvant radiotherapy while chemotherapy is restricted to patients not accessible for surgery and resistant to radionuclide therapy [8].

## 4 Conclusion

Non-functional retroperitoneal paragangliomas are rare group of tumors which are difficult to diagnose, owing to their silent and asymptomatic behavior, Once the diagnosis of a retroperitoneal paraganglioma has been made, surgical excision is required and is still the only curative approach.

## Ethical approval

I declare on my honor that the ethical approval has been exempted by my establishment.

## Sources of funding

None.

## Author contribution

Amal Hajri: writing the paper.

Ahmed Ballati: Corresponding author writing the paper.

Zakaria Essaidi: writing the paper.

Driss Errguibi: study concept.

Rachid Boufettal: study concept.

Saad Rifki El Jai: correction of the paper.

Farid Chehab: correction of the paper.

## Consent

Written informed consent for publication of their clinical details and/or clinical images was obtained from the patient.

## Registration of research studies

Not applicable.

## Guarantor

DOCTEUR AHMED BALLATI.

## Funding

None.

## Provenance and peer review

Not commissioned, externally peer-reviewed.

## Declaration of competing interest

The authors declare having no conflicts of interest for this article.
